# Delayed linkage to HIV care among asylum seekers in Quebec, Canada

**DOI:** 10.1186/s12889-019-8052-y

**Published:** 2019-12-16

**Authors:** Nadine Kronfli, Blake Linthwaite, Nancy Sheehan, Joseph Cox, Isabelle Hardy, Bertrand Lebouché, Alexandra de Pokomandy, Charles Frenette, Michel Roger, Marina B. Klein

**Affiliations:** 1 Department of Medicine, Division of Infectious Diseases and Chronic Viral Illness Service, Glen site, McGill University Health Centre, 1001 Decarie Boulevard D02.4110, Montreal, Quebec H4A 3J1 Canada; 2 Research Institute of the McGill University Health Centre, Montreal, Quebec Canada; 3 Faculté de Pharmacie, Université de Montréal, Montreal, Quebec Canada; 4 Département de Microbiologie, Infectiologie et Immunologie, Université de Montréal, Montreal, Quebec Canada; 5 Department of Family Medicine, McGill University, Montreal, QC Canada; 6 Centre Hospitalier de l’Université de Montréal, Montreal, Quebec Canada; 7 CIHR Canadian HIV Trials Network, Vancouver, British Columbia Canada

**Keywords:** HIV cascade of care, Asylum seekers, Screening, Linkage to care, Viral suppression, HIV resistance

## Abstract

**Background:**

Migrants represent an increasing proportion of people living with HIV in many developed countries. We aimed to describe the HIV care cascade and baseline genotypic resistance for newly diagnosed asylum seekers referred to the McGill University Health Centre (MUHC) in Montreal, Quebec, Canada.

**Methods:**

We conducted a retrospective cohort study of patients linked to the MUHC from June 1, 2017 to October 31, 2018. We calculated the median time (days; interquartile range (IQR)) from: 1) entry into Canada to immigration medical examination (IME) (i.e. HIV screening); 2) IME to patient notification of diagnosis; 3) notification to linkage to HIV care (defined as a CD4 or viral load (VL) measure); 4) linkage to HIV care to combination antiretroviral therapy (cART) prescription; and 5) cART prescription to viral suppression (defined as a VL < 20 copies/mL). We reviewed baseline genotypes and interpreted mutations using the Stanford University HIV Drug Resistance Database. We calculated the proportion with full resistance to > 1 antiretroviral.

**Results:**

Overall, 43% (60/139) of asylum seekers were newly diagnosed in Canada. Among these, 62% were late presenters (CD4 < 350 cells/μl), 22% presented with advanced HIV (CD4 < 200 cells/μl), and 25% with high-level viremia (VL > 100,000 copies/ml). Median time from entry to IME: 27 days [IQR:13;55]; IME to notification: 28 days [IQR:21;49]; notification to linkage: 6 days [IQR:2;19]; linkage to cART prescription: 11 days [IQR:6;17]; and cART to viral suppression: 42 days [IQR:31;88]; 45% were linked to HIV care within 30 days. One-fifth (21%) had baseline resistance to at least one antiretroviral agent; the K103 N/S mutation was the most common mutation.

**Conclusions:**

While the majority of newly diagnosed asylum seekers were late presenters, only 45% were linked to care within 30 days. Once linked, care and viral suppression were rapid. Delays in screening and linkage to care present increased risk for onward transmission, and in the context of 21% baseline resistance, consideration of point-of-care testing and immediate referral at IME screening should be made.

## Background

Many developed countries have observed recent shifts in their HIV epidemics, with a disproportionate number of new HIV diagnoses occurring among migrant populations [1, 2]. Migrants are at increased risk of HIV acquisition for several reasons. While many migrants leave countries with generalized HIV epidemics, the risk remains high post-migration as they often remain sexually active within migrant communities, where the HIV prevalence is higher than that of the receiving country [3]. Furthermore, changes in sexual behaviours post-migration, in combination with HIV-related stigma and discrimination, and the social disparities that accompany migration, all contribute to a higher risk of HIV acquisition during migration [4–6]. As treatment as prevention efforts expand and local attempts to eliminate HIV intensify, migrant populations remain vulnerable as they are frequently mobile, uninsured or unengaged in HIV care, and seldom the target of local prevention and treatment efforts, thereby potentially becoming a main source of new HIV infections [6].

Asylum seekers, refugee claimants and refugees represent an increasing proportion of people living with HIV in Canada [7]. Since 2010, Canada has processed a growing number of asylum claims made by individuals from HIV-endemic countries; the number of claims doubled from 2016 with over 50,000 processed in 2017 and 2018 alone [8]. While the province of Ontario has been the preferential refuge for most asylum seekers, Quebec surpassed Ontario during the past two years having processed approximately 25,000 asylum claims per year [8]. This shift is likely due to the higher influx of Haitian and other French-speaking African asylum seekers from the United States, as well as a higher number of irregular or unauthorized ports of entry in Quebec, where the Safe Third Country Agreement between the United States and Canada does not apply [9]. Under this Agreement, requests for refugee protection are required to be made in the first country of arrival (either Canada or the United States) [9].

All asylum seekers and refugee claimants must undergo an immigration medical examination (IME) in either Canada or overseas, respectively, that is administered by Immigration, Refugees and Citizenship Canada (IRCC) [10]. The IME includes a mandatory HIV screening test for all individuals 15 years of age and older, as well as for those under the age of 15 with known risk factors [11]. Immigration medical doctors, who perform the IMEs, facilitate linkage to HIV care among those who test positive. HIV-related care is assumed by the Interim Federal Health Program (IFHP), which provides limited and temporary health care coverage for in- and out-patient hospital services, further laboratory and diagnostic testing, and the costs of provincially-approved medications including combination antiretroviral therapy (cART) for asylum seekers, refugee claimants and certain other groups [12]. Asylum seekers, refugee claimants and refugees are not denied entry or residency in Canada based on a positive HIV diagnosis.

Data from IRCC indicate that while the proportion of HIV-positive diagnoses among all IME applicants has been stable in recent years, the overall number of people migrating to Canada has increased and, thus, the number of HIV-positive migrants to Canada has also increased [13]. This suggests that there should be heightened efforts to increase timely access to HIV services among migrant groups. A similar trend has been seen in Montreal, Quebec in the last three years. In 2015–2016, people from HIV-endemic countries represented only 15–20% of all new HIV diagnoses, whereas in 2017, represented 51% (Personal communications with Dr. Sarah-Amélie Mercure, 2019 Jan 10). Conversely, the proportion of new HIV diagnoses among men who have sex with men decreased from over 60% in 2015–2016 to 42% in 2017 in Montreal.

The HIV care cascade describes successive health care steps specific to HIV infection, from diagnosis to linkage to care, to treatment uptake and viral suppression, that result in optimal health outcomes. Timely engagement in HIV care has important individual-level health benefits, results in decreased forward transmission, and is essential to meeting the UNAIDS 90–90-90 objectives [14]. While linkage to care has been previously defined as accessing HIV care within 90 days of diagnosis, the current goal as per the Centre for Disease Control and Prevention (CDC) is 30 days [15]. The HIV care cascade has rarely been studied among migrant populations and never among Canadian asylum seekers [16]. Our primary aim was to quantify time to each step in the HIV care cascade among asylum seekers referred for care in order to inform future targeted health promotion and intervention development. Our secondary aim was to determine baseline genotypic resistance among all referred patients.

## Methods

### Study population and data collection

The Chronic Viral Illness Service (CVIS) is a multidisciplinary clinic focused on the care of individuals with chronic viral infections, and is located at the McGill University Health Centre (MUHC) in Montreal, Quebec, Canada. In 2018, there were 1849 patients who were in active care at the CVIS for either HIV infection alone or concomitant with another viral infection such as hepatitis B or C. The MUHC is the primary HIV referral centre for > 75% of asylum seekers in Montreal.

All adult (18 years of age or older) patients living with HIV (with or without another viral co-infection) who were linked to HIV care at the CVIS between June 1, 2017 and October 31, 2018, and whose care was covered by the IFHP (i.e. asylum seekers), were included in this retrospective cohort study. Patients meeting the inclusion criteria were identified retrospectively by either: (1) direct written or verbal notification of patients to the principal investigator by other CVIS physicians; or (2) letters written by CVIS physicians to patients’ designated immigration doctors; or (3) review of the CVIS social workers’ lists of patients with IHFP coverage. Patients were censored on the date of transfer to another clinic or at their last clinic visit prior to November 2018. Patients were excluded if they were referred to the CVIS during the study period without the appropriate transfer of documents necessary to determine the primary HIV care cascade outcome measures (*n* = 5). This study was approved by the Research Institute of the McGill University Health Centre Research Ethics Board (MUHC 2019–5037).

The CVIS maintains a clinical database and electronic medical records, both of which were the sources of information for all variables of interest. Retrospective reviews of patients’ data were performed to collect baseline sociodemographic (age, sex, sexual orientation) and psychosocial (substance use including alcohol, comorbidities such as mental health conditions) information, patient migration trajectories (including entry date in Canada), HIV laboratory parameters including CD4 count and viral load (VL) at presentation, and baseline genotype testing (if VL > 400 copies/mL). The dates of IME screening and diagnosis notifications were obtained from documents from outside laboratories and designated immigration doctors, respectively. Given the retrospective nature of the study, patient consent was not obtained. Instead, authorization to access patient charts was obtained from the Director of Professional Services of the MUHC – Adult Sector.

### Outcome variables

Among newly diagnosed asylum seekers, the primary outcome measures were median time in days from: (1) entry into Canada to IME screening; (2) IME screening to patient notification of diagnosis; (3) patient notification to linkage to HIV care (defined as either a CD4 cell count or VL measure); (4) linkage to initiation of cART (defined as the date a prescription was made for cART); and (5) initiation of cART to viral suppression (defined as a VL < 20 copies/mL). The proportion of late presenters (CD4 count < 350 cells/μL), those with advanced HIV infection (CD4 count < 200 cells/μL), and high-level viremia (VL > 100,000 copies/mL) was calculated. Among asylum seekers who were previously diagnosed with HIV infection outside Canada, a similar cascade of care was determined; however, median time from entry into Canada to linkage to HIV care was the first calculated time interval. Asylum seekers with known HIV infection were referred directly to the MUHC through the Programme Régional d’Accueil et d’Intégration des Demandeurs d’Asile (PRAIDA), which has a mandate from the Ministère de la Santé et des Services Sociaux to meet the needs of asylum seekers throughout Quebec. The proportion of patients who were linked within 30, 60 and 90 days from IME screening (among newly diagnosed asylum seekers) and from arrival in Canada (among previously diagnosed asylum seekers) was determined. Retention in care at six months and one year was calculated as the proportion of patients who attended a clinic visit at least six and 12 months after entering care, respectively.

Baseline genotypes were reviewed and mutations were interpreted using the Stanford University HIV Drug Resistance Database (Stanford HIVdb) [17]. Secondary outcome measures included the proportion of patients with full resistance to at least one antiretroviral (reported per class: nucleoside reverse transcriptase inhibitors (NRTI), non-nucleoside reverse transcriptase inhibitors (NNRTI), protease inhibitors (PI) and integrase inhibitor (II)), and multidrug resistance (resistance to two or more drugs from different antiretroviral classes). Genotypic susceptibility scores (GSS) were calculated as the sum of the individual GSS for each antiretroviral agent in a patient’s prescribed cART regimen following linkage to HIV care [18]. Based on GSS calculations by Frentz et al., each agent was given a score based on the level of resistance assigned by the Stanford HIVdb: 0, 0.25, 0.50, 0.75, or 1 for high-level, intermediate, low-level, potential low-level, and susceptible, respectively [18]. A score of 3 or higher indicates a fully susceptible cART regimen. Only genotypes taken prior to or up to eight days after the start of a given cART regimen were considered when calculating GSS [18]. The proportion of patients with M184 V/I, K103 N/S, and K65R mutations, as well as the presence of thymidine analogue mutations (TAMs) were also reported. The proportion of patients with virologic failure (defined as the inability to achieve and maintain a VL < 200 copies/mL after 6 months of antiretroviral therapy) and the development of new resistance mutations were evaluated [19].

### Statistical analysis

Summary statistics, medians and interquartile ranges (IQR) or counts and proportions, were calculated to describe the sample. All analyses were performed in R-3.5.1.

## Results

### Patient characteristics

A total of 139 asylum seekers were included with a median follow-up time of 9.7 [5.1, 12.2] months. Patient sociodemographic and clinical characteristics at initial visit are presented in Table 1. Median age was 38 years, 63% were female, and 86% self-identified as heterosexual. A minority were co-infected with either hepatitis B or C while one-third had positive tuberculin skin tests. Over one-third of patients were of Haitian ethnicity and approximately one-quarter were Nigerian; 84% of all asylum seekers transited through the United States before entering Canada.

**Table 1:** Baseline characteristics of the study sample

	Overall (*n* = 139)	First diagnosed in Canada (*n* = 60)	First diagnosed before arrival to Canada (*n* = 79)
Age (median [IQR])	38 [33; 45]	37 [33; 44]	39 [33; 47]
Sex			
Female	88 (63%)	31 (52%)	57 (72%)
Male	51 (37%)	29 (48%)	22 (28%)
Sexual orientation			
Heterosexual	120 (86%)	51 (85%)	69 (87%)
LGBTQ	19 (14%)	9 (15%)	10 (13%)
Country of origin			
Africa			
Nigeria	33 (24%)	12 (20%)	21 (27%)
Other	54 (39%)	19 (31%)	36 (46%)
Latin America			
Haiti	48 (34%)	29 (48%)	19 (24%)
Other	4 (3%)	0 (0%)	3 (4%)
History of sexual- or gender-based violence	57 (41%)	25 (42%)	32 (41%)
Rape	35 (25%)	16 (26%)	19 (24%)
CD4 at presentation in Canada, cells/μl (median, range, [IQR])	415, 7–1221, [275; 656.5]	307, 11–811, [221; 401]	574, 7–1221, [382; 756]
CD4 < 200	22 (16%)	13 (22%)	9 (11%)
CD4 < 350	52 (37%)	37 (62%)	15 (19%)
Baseline viral load, copies/ml (median, range, [IQR])	1970, < 20 - > 1 million, [< 20; 41,313]	32,349, < 20 - > 1 million, [7890; 100,594]	< 20, < 20 - > 1 million, [< 20; 181]
OI at presentation	1 (1%)	1 (2%)	0 (0%)
Requiring primary prophylaxis for OI	23 (16%)	12 (20%)	10 (13%)
cART regimens, baseline			
On cART	61 (44%)	0 (0%)	61 (77%)
3rd agent			
NNRTI	35 (57%)	0 (0%)	35 (57%)
PI	5 (8%)	0 (0%)	5 (8%)
II	16 (26%)	0 (0%)	16 (26%)
Unknown	4 (7%)	0 (0%)	4 (7%)
Single tablet regimens	41 (67%)	0 (0%)	41 (67%)
cART regimens, end of study period			
On cART	133 (96%)	58 (97%)	75 (95%)
3rd agent			
NNRTI	8 (6%)	1 (2%)	7 (9%)
PI	2 (2%)	0 (0%)	2 (3%)
II	123 (92%)	57 (98%)	66 (88%)
Single tablet regimens	103 (77%)	42 (72%)	61 (81%)
TST			
Positive	47 (34%)	16 (27%)	31 (39%)
Negative	84 (60%)	41 (68%)	43 (54%)
Not done/missing	8 (6%)	3 (5%)	5 (6%)
Co-infection with HBV	8 (6%)	4 (7%)	4 (5%)
Co-infection with HCV	1 (0.7%)	0 (0%)	1 (1%)

*ARV* antiretroviral, *cART* combination antiretroviral therapy, *HBV* hepatitis B virus, *HCV* hepatitis C virus, *II* integrase inhibitor, *IQR* interquartile range, *LGBTQ* lesbian, gay, bisexual, transgender, and/or queer, *NNRTI* non-nucleoside reverse transcriptase inhibitor, *OI* opportunistic infection, *PI* protease inhibitor, *TST* tuberculin skin test

Overall, 43% of asylum seekers were newly diagnosed in Canada with a median CD4 count of 307 cells/μl and VL of 32,349 copies/mL at presentation. Among these, 62% were late presenters (CD4 < 350 cells/μl), 22% presented with advanced HIV (CD4 < 200 cells/μl), and 25% presented with high-level viremia (VL > 100,000 copies/ml); one patient was an elite controller. Primary prophylaxis for HIV-associated opportunistic infections (OI) was prescribed for 20% of newly diagnosed asylum seekers. Among those previously diagnosed outside Canada, 77% were on cART at initial presentation; median CD4 count and VL were 621 cells/μl and < 20 copies/mL, respectively among those on cART, and 242 cells/μl and 41,190 copies/mL, respectively among those not on cART. However, 29% had detectable VLs at presentation (including six of the 61 persons taking cART) and 13% required primary prophylaxis. Opportunistic infections were rare in both groups; one newly diagnosed asylum seeker was hospitalized for 11 days for cerebral toxoplasmosis.

### HIV cascade of care

Among the 139 patients who were linked to care, 96% were prescribed cART; five had not yet seen a physician by the end of the study period and one was an elite controller. The majority of patients (77%) were prescribed a single-tablet regimen and an integrase inhibitor was the most commonly (92%) prescribed third agent. Among those on cART and in care, 87% (87/100) were virally-suppressed at six months and 97% (36/37) at one year after linkage, respectively; the proportion with virologic failure was 4% (4/100) at 6 months. All patients were retained in care at six months and one year.

**Fig. 1:**
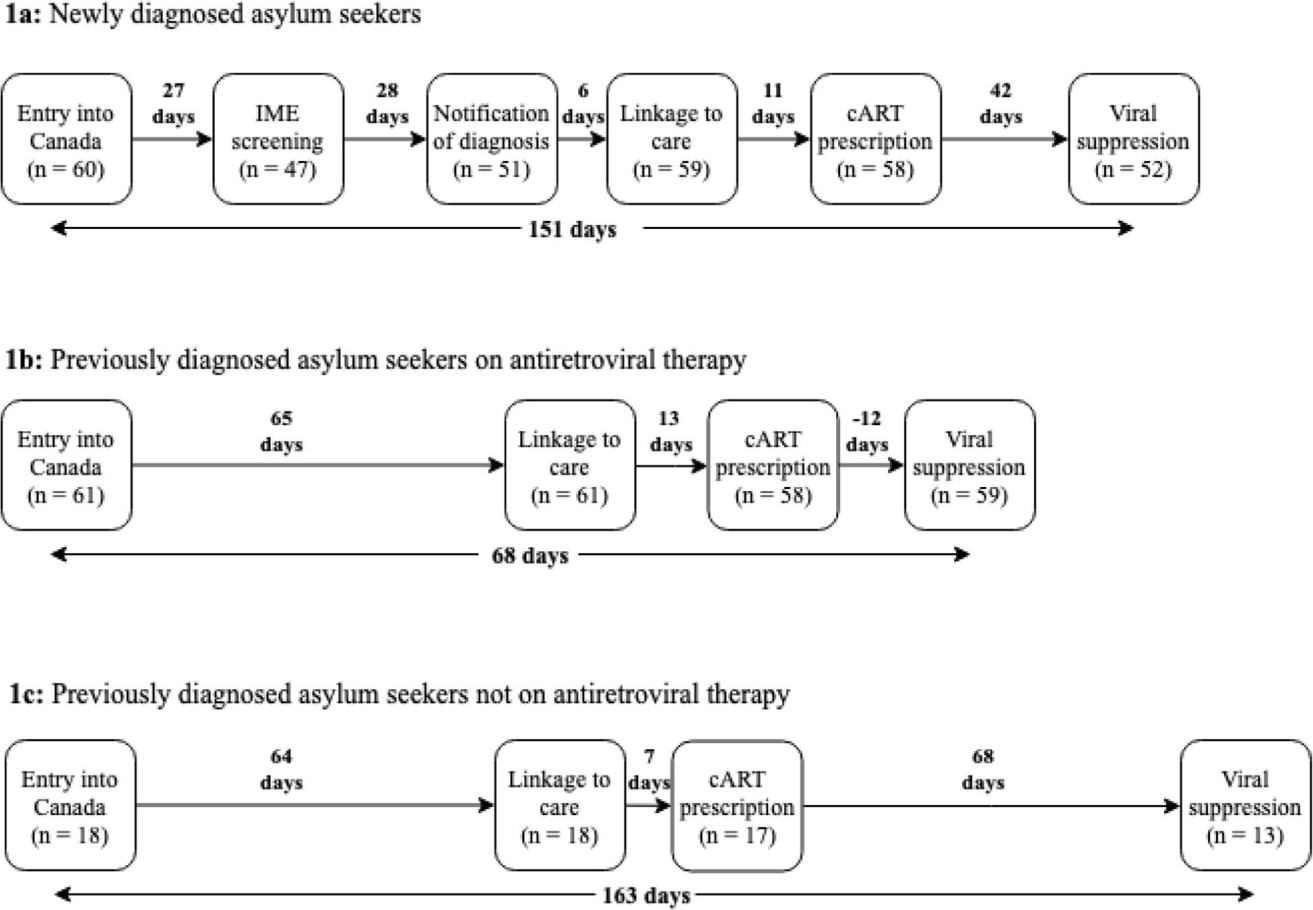
HIV cascades of care among newly diagnosed asylum seekers **1a**, previously diagnosed asylum seekers on antiretroviral therapy **1b**, and previously diagnosed asylum seekers not on antiretroviral therapy **1c**

Among newly diagnosed asylum seekers (*n* = 60), median time from entry into Canada to IME screening: 27 days [IQR: 13; 55]; IME screening to notification of diagnosis: 28 days [IQR: 21; 49]; notification to linkage: 6 days [IQR: 2; 9]; linkage to cART prescription: 11 days [IQR: 6; 17]; and cART prescription to viral suppression: 42 days [IQR: 31; 88] (Fig. 1a). Median time from entry to linkage to care was 80 days [IQR: 57; 102]. Overall, 45% of new diagnoses were linked within 30 days, 80% within 60 days and 90% within 90 days from HIV screening. Median time from entry into Canada to viral suppression was 151 days [IQR: 121; 216]. We did not have data regarding IME screening or notification of diagnosis among 22% (13/60) and 15% (9/60) of newly diagnosed asylum seekers, respectively.

Among previously diagnosed asylum seekers who were on cART at presentation (*n* = 61), time from entry to linkage: 65 days [IQR: 45; 86]; linkage to cART prescription: 13 days [IQR: 5; 24], and cART prescription to viral suppression: − 12 days [IQR: − 21; − 4] (Fig. 1b). Among these, 90% were virally suppressed at presentation. Overall, 20% of previous diagnoses on cART at presentation were linked within 30 days, 48% within 60 days and 79% within 90 days from entry into Canada. Median time from entry into Canada to viral suppression was 68 days [IQR: 48; 91].

Among previously diagnosed asylum seekers who were not on cART at presentation (*n* = 18), time from entry to linkage: 64 days [IQR: 22; 102]; linkage to cART prescription: 7 days [IQR: 4; 10], and cART prescription to viral suppression: 68 days [IQR: 28; 89] (Fig. 1c). Overall, 28% of previous diagnoses not on cART at presentation were linked within 30 days, 44% within 60 days and 67% within 90 days from entry into Canada. Median time from entry into Canada to viral suppression was 163 days [IQR: 141; 185].

### Antiretroviral resistance

Baseline genotypes were available for 95% (57/60) of newly diagnosed asylum seekers and 29% (23/79) of previously diagnosed asylum seekers (i.e. all individuals who were viremic at presentation) (Table 2). Among newly diagnosed asylum seekers, 21% had baseline resistance to at least one antiretroviral agent while 35% of previously diagnosed asylum seekers had baseline ARV resistance. Baseline resistance to at least one NNRTI was the most commonly observed resistance (with 12% of newly diagnosed asylum seekers exhibiting K103 N/S mutations; of these, 5/7 were from Latin America and 2/7 were from Africa), followed by NRTI resistance. M184 V/I, K65R and TAMs were uncommon, as was multidrug resistance. One newly diagnosed patient, who was unknowingly prescribed functional monotherapy, developed full integrase inhibitor resistance (N155H, Q148R, S147G, E138K, and E92Q mutations). The median GSS was 3 [IQR 3; 3] for the entire study population. Among the four patients with virologic failure, one developed two new resistance mutations (M184 V and N155H).

**Table 2:** Baseline antiretroviral resistance

	Overall (*n* = 139)	First diagnosed in Canada (*n* = 60)	First diagnosed before arrival to Canada (*n* = 79)
HIV Genotype available	80 (58%)	57 (95%)	23 (29%)
Baseline antiretroviral resistance^a^			
No	60 (75%)	45 (79%)	15 (65%)
Yes	20 (25%)	12 (21%)	8 (35%)
NRTI resistance	6 (8%)	2 (4%)	4 (17%)
NNRTI resistance	17 (21%)	12 (21%)	5 (22%)
PI resistance	0 (0%)	0 (0%)	0 (0%)
II resistance	1 (1%)	1 (2%)	0 (0%)
Multidrug resistance	3 (4%)	2 (4%)	1 (4%)
M184 V/I mutation	3 (4%)	2 (4%)	1 (4%)
K103 N/S mutation	11 (14%)	7 (12%)	4 (17%)
K65R mutation	0 (0%)	0 (0%)	0 (0%)
TAMs	1 (1%)	1 (2%)	0 (0%)

^a^The denominator for these proportions is the number of patients with available genotypic data (i.e. 80 for overall, 57 for first diagnosed in Canada, and 23 for first diagnosed before arrival to Canada)

*II* integrase inhibitor, *NRTI* nucleoside reverse transcriptase inhibitor, *NNRTI* non-nucleoside reverse transcriptase inhibitor, *PI* protease inhibitor, *TAMs* Thymidine analogue mutations

## Discussion

Our study retrospectively identified that the major bottleneck along the HIV cascade of care for Canadian asylum seekers was linkage to care. We observed that even in a system with a clear care pathway, a minority (45%) of newly diagnosed asylum seekers were linked to care within 30 days – the current CDC standard [15]. The majority (62%) of newly diagnosed asylum seekers presented with a CD4 count of < 350 cells/μl, and an important minority (22%) with advanced HIV infection (CD4 count < 200 cells/μl) necessitating primary OI prophylaxis, which not only reflects findings from other European studies, but underscores the importance of timely linkage for individual health benefits [20–22]. That said, we found that rapid cART initiation once linked was possible, that retention was 100% at six and 12 months, and that viral suppression was achieved for almost all. We also observed that while the majority (77%) of asylum seekers with known HIV infection arrived with a supply of cART, nearly one-third had a detectable HIV RNA at presentation, including 10% of those on cART. Even longer delays to linkage to care were observed among those with a prior HIV diagnosis. Delays in screening and care can increase the likelihood of onward transmission, and in the context of non-trivial baseline resistance (~25% of the overall population), expediting screening and linkage to care to prevent transmission of resistant viruses becomes even more important.

Accelerating linkage to HIV care for newly diagnosed asylum seekers should be considered a priority going forward. The current system in Canada requires that all asylum seekers entering the country schedule an IME within 30 days of arrival [23]. In fact, we observed that the median time to the IME following arrival in Canada among newly diagnosed asylum seekers was 27 days, meaning that approximately 50% received their IMEs after the IRCC cut-off. The reasons for this delay are unknown, but as asylum seekers are exempt from IME fees, this may be related to a lack of knowledge, a low perception of risk, competing priorities such as housing, or a shortage of immigration medical doctors [23, 24]. Currently, HIV testing required as part of the IME is not a point-of-care service; rather, all bloodwork is routinely done through community-based laboratories. Consequently, we observed an additional 28-day delay from the IME to a patient’s notification of his/her new HIV diagnosis. Thereafter, the median time to initiation of cART following notification was only 17 days. These findings underscore that the major bottleneck along the HIV care cascade were proximal to disease notification, and while rapid cART initiation was possible, suggest that newly diagnosed asylum seekers were at risk of contributing to forward HIV transmission for a median of 72 days prior to the initiation of antiretrovirals. Knowing that migration itself can enhance sexual behaviours, particularly among men who have sex with men, these delays could potentially have important consequences for public health [4, 5]. The importance of identifying transmission clusters through phylogenetic analyses among this sub-population thus becomes particularly relevant. The results of such analyses could then be used to advocate for either point-of-care HIV testing at the time of entry or as part of the IME, moving towards a “test-and-treat” model of care for this population.

Previously diagnosed asylum seekers who arrived in Canada without a supply of antiretrovirals are another priority group for accelerated linkage to HIV care. While these individuals represented only 23% of those with previously known HIV, their immune systems appeared to be more compromised than newly diagnosed asylum seekers; their median CD4 count was 242 cells/μl vs. 307 cells/μl, respectively. Individuals with known HIV are expected to declare their status at the border, at which point, they are referred to AIDS service organizations (ASOs) for accelerated linkage to care. This information (i.e. disclosure of status and referrals to ASOs) was not extracted by our retrospective chart reviews; however, there was a median of 71 days between entry into Canada and cART re-initiation, with the major bottleneck similarly occurring from entry to linkage (64 days). This suggests that disclosure may have rarely occurred at the time of border crossing, likely due to the fear of being denied entry into Canada. This also implies that there may be a role for the improved provision of information at the time of entry into Canada, including allaying fears regarding the denial of people living with HIV, and the availability of free medical care to reduce interruptions of therapy.

We found that the overall frequency of drug resistance among newly diagnosed asylum seekers was 21% – 1.5-fold higher than provincial (13.1%) and national (13.9%) levels [25, 26]. It is unclear whether the higher observed resistance rates represent true transmitted resistance, prior undisclosed treatment, or exposure to nevirapine in the context of prevention of mother-to-child transmission (MTCT) efforts. The frequency of both K103 N/S and M18 V/I resistance mutations was three-fold higher in our newly diagnosed population compared to provincial data. The K103 N/S mutation predominated, reflecting that efavirenz and nevirapine continue to be widely used in many developing countries. In fact, among the seven newly diagnosed individuals with a K103 N/S mutation, three were women with living children, suggesting that prior exposure to nevirapine for MTCT may have been possible. That said, the frequency of K103 N/S resistance mutations among treatment-experienced patients was identical to provincial data (17%) [25]. Conversely, we observed a much lower frequency (4.3%) of M184 V/I resistance mutations among treatment-experienced patients than the provincial frequency of 30%, which may represent truly lower baseline rates or an underestimation due to M184 V/I’s potential to be archived. Our findings imply that, although one in five newly diagnosed asylum seekers shows evidence of drug resistance, withholding cART while awaiting baseline genotypes is likely unnecessary. Providers should initiate a first-line integrase-based cART regimen as soon as possible, recognizing that M184 V/I resistance mutations are rare (or possibly underestimated) and that NNRTIs should be avoided (as per current guidelines) [27]. The baseline genotypic susceptibility scores of 3 corroborate this; newly diagnosed patients were prescribed fully functional regimens even in the absence of available baseline genotypes. That said, baseline genotypic testing (including integrase) should still be done on everyone especially as first line regimens in developing countries are evolving to contain integrase inhibitors.

Our retrospective cohort study represents one of the first in Canada and globally to quantify the HIV care cascade among asylum seekers. Although our results are likely generalizable to asylum seekers in other Canadian provinces as identical care pathways exist (i.e. IME within 30 days of arrival, linkage via immigration medical doctors, etc.) throughout Canada, our analysis is limited to asylum seekers who are linked to care in a tertiary care centre with specialized HIV services. Our study has other limitations. First, there are important delays in the HIV care cascade that we were not able to accurately quantify. These include the time from cART prescription to cART initiation, as well as the true time to viral suppression given inconsistent VL measurements. Second, we were not powered to determine correlates associated with delays along the HIV care cascade although many sociodemographic (ethnicity, gender, sexual orientation, etc.) and psychosocial (financial or housing insecurity, HIV-related stigma, etc.) factors may have played a role as other studies have shown [1, 23]. For example, while 40% of the study population reported a history of sexual- or gender-based violence, and certain ethnocultural groups reported worse financial strain than others, due to the retrospective nature of this study, validated tools were not used to assess these correlates. Future qualitative studies could add insight into how best to reduce delays in linkage to HIV care for asylum seekers.

## Conclusions

Migrant populations represent an increasing proportion of people living with HIV in many developed countries such as Canada. Our study has demonstrated that even in a system with a clearly defined care pathway, there is a need to expedite screening and linkage to HIV care for asylum seekers. This may be in the form of either point-of-care HIV testing at the time of entry or as part of the IME, moving towards a “test-and-treat” model of care. With globalization and current trends in migration, prioritizing migrant populations in HIV care engagement will not only have important individual-level health outcomes, but will benefit society as a whole.

## Data Availability

The data generated and/or analysed during the current study are not publicly available due to privacy regulations, but de-identified data are available from the corresponding author on reasonable request.

## Abbreviations

cART: Combination antiretroviral therapy
CDC: Centre for Disease Control and Prevention
CVIS: Chronic Viral Illness Service
GSS: Genotypic susceptibility score
HIV: Human immunodeficiency virus
HIVdb: HIV database
IFHP: Interim Federal Health Program
II: Integrase inhibitor
IME: Immigration medical exam
IQR: Interquartile range
IRCC: Immigration, Refugees and Citizenship Canada
MTCT: Mother-to-child transmission
MUHC: McGill University Health Centre
NNRTI: Non-nucleoside reverse transcriptase inhibitor
NRTI: Nucleoside reverse transcriptase inhibitor
OI: Opportunistic infection
PI: Protease inhibitor
PRAIDA: Programme Régional d’Accueil et d’Intégration des Demandeurs d’Asile
TAMs: Thymidine analogue mutations
VL: Viral load
